# Eosinophil and IFN-γ associated with immune-related adverse events as prognostic markers in patients with non-small cell lung cancer treated with immunotherapy

**DOI:** 10.3389/fimmu.2023.1112409

**Published:** 2023-03-06

**Authors:** Wei-Ting Hu, Qiurui Zhang, Ze Zhang, Xuan He, Min Zhou, Yi Guo, Xiaofei Wang

**Affiliations:** ^1^ Department of Pulmonary and Critical Care Medicine, Ruijin Hospital, Shanghai Jiao Tong University School of Medicine, Shanghai, China; ^2^ Institute of Respiratory Diseases, Shanghai Jiao Tong University School of Medicine, Shanghai, China; ^3^ Shanghai Key Laboratory of Emergency Prevention, Diagnosis and Treatment of Respiratory Infectious Diseases, Shanghai, China; ^4^ Department of Pharmacy, Ruijin Hospital, Shanghai Jiao Tong University School of Medicine, Shanghai, China

**Keywords:** non-small cell lung cancer(NSCLC), immune checkpoint inhibitor(ICI), immune related adverse event(irAE), eosinophil count, interferon-gamma(IFN-γ)

## Abstract

**Objectives:**

Immune checkpoint inhibitors (ICIs) alone or combined with other antitumor agents are largely used in lung cancer patients, which show both positive effects and side effects in particular subjects. Our study aims to identify biomarkers that can predict response to immunotherapy or risk of side effects, which may help us play a positive role and minimize the risk of adverse effects in clinical practice.

**Methods:**

We retrospectively collected data from patients with advanced non-small cell lung cancer (NSCLC) treated with ICIs at our center. Patients who received initial ICI therapy for >1 year without progression of disease were classified as long-term treatment (LT) group, while others were classified as the non-long-term treatment (NLT) group. Multivariate logistic analysis was performed to identify independent risk factors of progression-free survival (PFS) and immune-related adverse events (irAEs).

**Results:**

A total of 83 patients (55.7%) had irAEs. The median PFS for patients in grades 1–2 of irAEs vs. grades 3–4 vs non-irAEs groups was (undefined vs. 12 vs. 8 months; *p* = 0.0025). The 1-year PFS rate for multisystem vs. single vs. non-irAE groups was 63%, 56%, and 31%, respectively. Signal transduction of inflammatory cytokines improves clinical prognosis through immunomodulatory function, but the benefit is also limited by the resulting organ damage, making it a complex immune balance. Serum biomarkers including EOS% of ≥ 1.15 (HR: 8.30 (95% CI, 2.06 to 33.42); *p* = 0.003) and IFN-γ of ≥ 3.75 (HR: 5.10 (95% CI, 1.29 to 20.15), *p* = 0.02) were found to be predictive for irAEs.

**Conclusion:**

EOS% of ≥1.15% and IFN-γ of ≥3.75 ng/L were considered peripheral-blood markers for irAEs and associated with improved clinical outcomes for immunotherapy in patients with advanced NSCLC.

## 1 Introduction

As a result of recent advances in immune-checkpoint inhibition therapy (ICI), patients with metastatic non-small cell lung cancer (NSCLC) now have more treatment options, including ICI alone or in combination with chemotherapy. Antiangiogenic therapy has shown an unprecedented duration of response in some patients. For example, up to 16% of NSCLC stage IV patients treated with nivolumab in the second line and 31.9% of patients treated with pembrolizumab in the first line survived 5 years (1–3). However, there is still a certain population of patients who do not respond to treatment or even suffer from immune-related adverse events (irAEs). irAEs are any unfavorable and unintended sign, symptom, or disease that is temporally associated with the use of ICIs. Based on the latest research, ICI therapy activates T cells in the immune system to fight against cancer cells, which may also attack healthy tissues because of common antigens (4), leading to various adverse events, such as skin rashes, colitis, hepatitis, pneumonia, etc. (5). Tissue-based programmed cell death-ligand 1 (PD‐L1) expression is the main criterion for the prediction of ICI treatment and has been shown to correlate with the efficacy of PD‐1/PD‐L1 blockade therapy in NSCLC (6, 7). Real-world experiences have shown that ICIs are often prescribed in combination with different agents, which may further reduce the predictive value of PD-L1 and complicate the toxicity of immunotherapy. Therefore, it is important to identify reliable predictors of clinical benefits from ICI-based therapy as well as risk factors for irAEs. The purpose of this study is to investigate the association between baseline clinical characteristics and response to different strategies using ICIs in patients with advanced NSCLC and assess the risk factor in baseline peripheral blood that correlated with irAEs.

## 2 Method

### 2.1 Subjects

**Figure 1:**
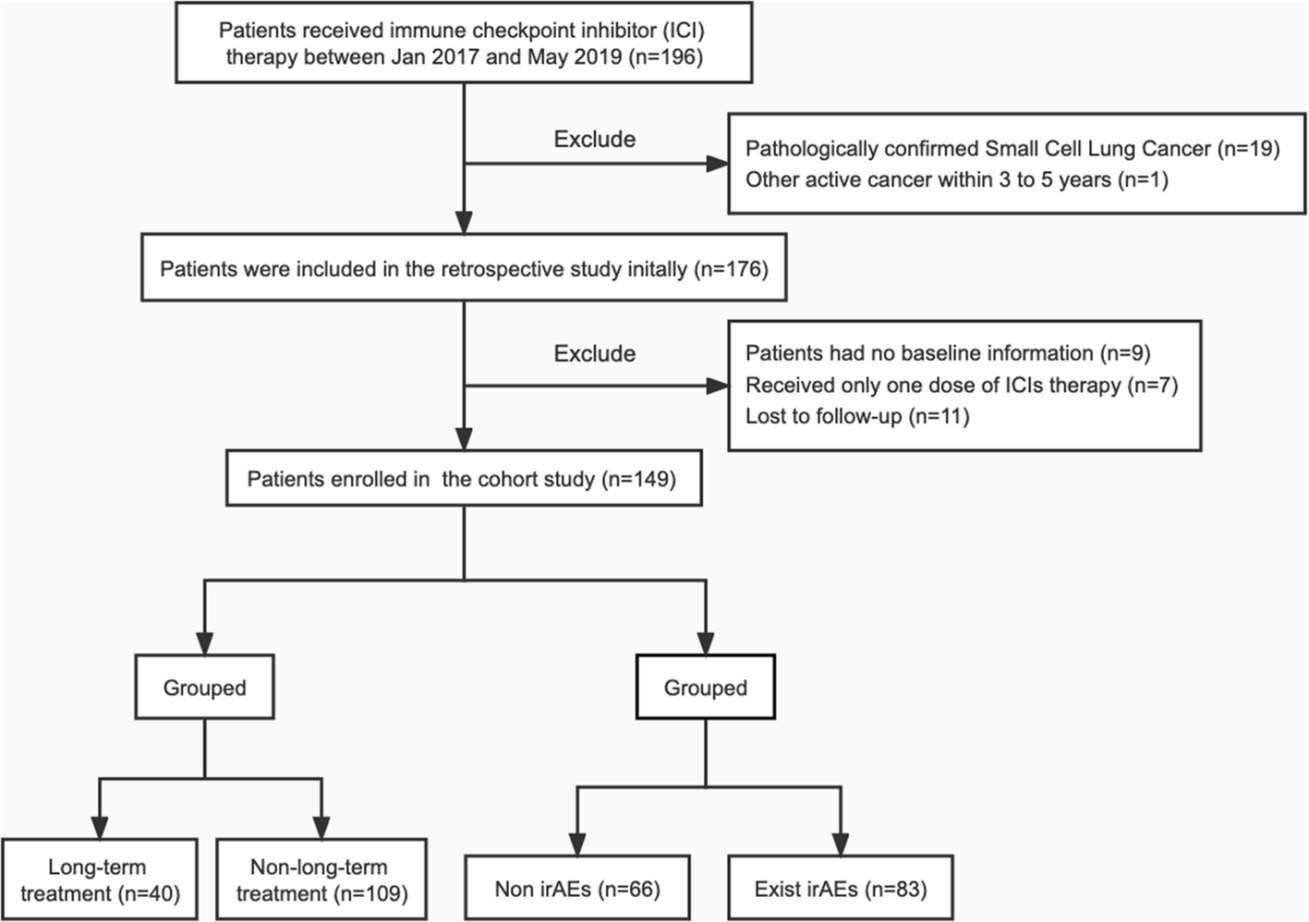
Flow chart of the study design. ICI, immune checkpoint inhibitor; irAEs, immune-related adverse events.

Between January 2017 and May 2019, 149 patients with NSCLC who were treated at the Department of Respiratory Diseases and Critical Care Medicine at Ruijin Hospital (Shanghai, China) were retrospectively reviewed. Inclusion criteria were the availability of a clinical database, adequate follow‐up, and treatment with ICIs according to clinical practice. The flowchart of the study design is presented in **Figure 1**.

Patients’ data collected at baseline included patients’ demographics, Eastern Cooperative Oncology Group performance status (ECOG PS) at the initiation of ICI, smoking history, and comorbidities. Tumor data collected included histology and molecular results for EGFR, ALK, MET, HER‐2, K‐RAS, ROS‐1, BRAF, RET, and PD‐L1 status, when available. Radiological imaging performed before and during the process of ICI treatment was systematically reviewed. ICI-related toxicity data were collected from treating physicians during and after treatment. Baseline blood count data were defined as the most recent blood count within 1 week before ICI initiation. This study was approved by the Ethics Committee of Ruijin Hospital.

### 2.2 Study assessments

Patients who received initial ICI therapy for >1 year without progressive disease were classified as the long-term treatment (LT) group; others were classified as the non-long-term treatment (NLT) group (8). Drug efficacy was assessed every 8–12 weeks based on the Response Evaluation Criteria in Solid Tumors (RECIST, version 1.1) by computed tomography (CT) scan (9). Progression-free survival (PFS) was defined as the first day of immunotherapy to the date of disease progression. IrAE was diagnosed by multidisciplinary adjudication, which is based on clinical manifestations and objective evidence. For inclusion in the present study, the irAE had to have a certain or probable causal association with the anti-PD-1 or anti-PD-L1 inhibitor as assessed on the World Health Organization Uppsala Monitoring Centre scale. All irAEs were reported and classified using the National Cancer Institute Common Terminology Criteria for Adverse Events, version 4.03. The following data were reviewed: characteristics of the ICIs and any concomitant treatments received; clinical and laboratory characteristics of the patients, characteristics of the irAEs, medications administered to treat the irAEs, and the outcome of the irAEs.

### 2.3 Statistical analysis

All quantitative parameters are expressed as the means ± standard deviations (SDs) or median and interquartile (25th–75th percentiles), as appropriate. The Kolmogorov–Smirnov tests were performed to test for a normal distribution. Differences in clinical characteristics among different groups were evaluated using *t-*tests and Mann-Whitney *U* tests for continuous variables, and the chi-squared test and Fisher’s exact probability method were used for categorical variables. PFS curves were calculated using the Kaplan–Meier method, and the log-rank test was employed to assess differences. Cox regression models were applied to find independent indicators associated with PFS. Logistic regression analysis was applied to explore the correlation between peripheral-blood markers and the onset of irAEs. Factors that were statistically significant in the univariate analysis were incorporated into the multivariate analysis. Receiver operating characteristic (ROC) curves and the area under the curve (AUC) were employed to evaluate the ability of the peripheral-blood markers to predict the irAEs, as well as its cutoff value. SPSS Version 25.0 statistical software (IBM Corporation, Armonk, NY, USA) was used for all the statistical tests. A *p*-value of < 0.05 was considered statistically significant.

## 3 Results

### 3.1 Patient demographics and disease characteristics

This study included 149 patients with advanced NSCLC, 83 of whom were ≥ 65 years old and 66 were <65 years old. Most of them (116/149, 77.9%) were male patients. Of the 149 participants, 93 were former or current smokers. Among these 45 squamous and 104 nonsquamous lung cancer patients, most had an ECOG performance status of 0–1 (133/149, 89.3%). There were no or undetected sensitive gene mutations in 96 patients (96/149, 64.4%). There were enough tissue for PD-L1 analysis in 101 patients (101/149, 67.8%), 72 of whom (72/101, 71.3%) were PD-L1 positive (44 patients ≥ 50%, 28 patients ≥ 1%), while the remaining 29 (29/101, 28.7%) were negative (<1%). All patients were treated with ICIs, either as monotherapy (35/149, 23.5%) or in combination therapy (114/149, 76.5%), as shown in **Supplementary Table S1**. PD-1 inhibitors were accepted as first-line treatment by 69 patients (69/149, 46.3%). There were irAEs in 55.7% of the cases (83/149). Baseline demographics and disease characteristics are summarized in **Table 1**.

**Table 1:** Baseline characteristics of a cohort of 149 patients with NSCLC.

Characteristic	Total (*N* (%))
Age	
≥65	83 (55.7)
<65	66 (44.3)
Sex	
Female	33 (22.1)
Male	116 (77.9)
Smoking status	
Smoker	93 (62.4)
Nonsmoker	56 (37.6)
Histology	
Squamous	45 (30.2)
Nonsquamous	104 (69.8)
ECOG	
0–1	133 (89.3)
≥2	16 (10.7)
PD-L1 expression	
PD-L1 ≥50%	44 (29.5)
PD-L1 1–49%	28 (18.8)
PD-L1 <1%	29 (19.5)
Unknown	48 (32.2)
Drive mutations	
EGFR/ALK mutation	25 (16.8)
Non-EGFR/ALK	28 (18.8)
KRAS mutation	15 (10.1)
Wild type	59 (39.6)
Unknown	37 (24.8)
Therapeutic lines	
First-line	69 (46.3)
Second-line and above	80 (53.7)
Therapeutic schedule	
Monotherapy	35 (23.5)
Combined treatment	114 (76.5)
irAEs	
Exist irAEs	83 (55.7)
Non-irAEs	66 (44.3)

PD-L1, programmed cell death-ligand 1; EGFR, epidermal growth factor receptor; ALK, anaplastic lymphoma kinase; KRAS, Kirsten rat sarcoma viral oncogene homolog; irAEs, immune-related adverse events.

### 3.2 Comparison of characteristics between NLT and LT

The median PFS (mPFS) was 11 months in the overall population, with a median follow-up time of 13.0 months (95% CI: 11.0–15.0). First-line ICI therapy (67.5% vs. 38.5%; *p* = 0.002) and the presence of irAEs (77.5% vs. 47.7%; *p* = 0.001) were found to be associated with longer PFS. Furthermore, patients treated with ICIs for combined treatment (*p* = 0.055) and patients without EGFR/ALK-driven mutations (*p* = 0.066) were predicted to benefit longer from ICI therapy.

However, patients’ age, gender, tobacco use, ECOG score, and level of PD-L1 were found to be no different between LT and NLT groups. A previous study showed that the baseline feature of a high eosinophil count was associated with a better clinical outcome (10). In our study, neither baseline peripheral-blood eosinophil count (or percentage) nor blood CD3/4/8 level and cytokine levels (IL-6, IL-8, IL-10, IL-1β, TNF-α, IFN-γ) were found to be significantly different between LT and NLT patients treated with ICIs (**Table 2**).

**Table 2:** Characteristics of patients with NLT or LT.

Characteristic	NLT	LT	*p*-value
Number	109	40	–
Age (mean ± SD)	65 ± 10	65 ± 9	0.349
Female (*N* (%))	26 (23.9)	7 (17.5)	0.408
Smoker (*N* (%))	65 (59.6)	28 (70.0)	0.247
Squamous (*N* (%))	31 (28.4)	14 (35.0)	0.440
ECOG ≥2 (*N* (%))	13 (11.9)	3 (7.5)	0.329
PD-L1 ≥50% (*N* (%))	29 (39.7)	15 (53.6)	0.209
PD-L1 <1% (*N* (%))	21 (28.8)	8 (28.6)	0.984
EGFR/ALK mutations (*N* (%))	22 (20.2)	3 (7.5)	0.066
KRAS mutation (*N* (%))	12 (11.0)	3 (7.5)	0.760
First-line ICIs (*N* (%))	42 (38.5)	27 (67.5)	0.002^*^
Combined treatment (*N* (%))	79 (72.5)	35 (87.5)	0.055
Exist irAEs (*N* (%))	52 (47.7)	31 (77.5)	0.001^*^
Neu%	68.2 ± 9.7	67.7 ± 11.0	0.783
L%	21.3 ± 8.2	20.2 ± 9.3	0.476
EOS%	2.6 ± 2.7	3.4 ± 4.8	0.219
Neu (×10^9^/L)	5.0 ± 2.7	5.3 ± 3.1	0.869
L (×10^9^/L)	1.4 ± 0.6	1.3 ± 0.6	0.627
NLR	4.1 ± 2.8	5.0 ± 4.6	0.651
EOS (×10^9^/L)	0.2 ± 0.2	0.3 ± 0.5	0.374
CRP (mg/L)	21.4 ± 39.5	49.3 ± 83.9	0.156
CD3	918.0 ± 434.2	900.6 ± 391.1	0.845
CD4	531.4 ± 287.1	486.3 ± 254.3	0.574
CD8	349.3 ± 221.6	376.9 ± 176.7	0.213
NK	312.4 ± 208.9	285.8 ± 192.5	0.696
IL-6 (ng/L)	31.8 ± 110.0	23.3 ± 32.9	0.175
IL-8 (ng/L)	55.7 ± 73.4	61.3 ± 58.6	0.344
IL-10 (ng/L)	4.1 ± 2.6	4.9 ± 5.3	0.777
IL-1β (ng/L)	6.4 ± 5.2	6.2 ± 6.8	0.460
TNF-α (ng/L)	5.2 ± 4.5	5.2 ± 5.2	0.949
IFN-γ (ng/L)	6.3 ± 8.1	7.4 ± 16.8	0.397

^*^p < 0.05.

NLT, non-long-term treatment; LT, long-term treatment; PD-L1, programmed cell death-ligand 1; EGFR, epidermal growth factor receptor; ALK, anaplastic lymphoma kinase; KRAS, Kirsten rat sarcoma viral oncogene homolog; irAEs, immune-related adverse events; ICIs, immune checkpoint inhibitors; Neu, neutrophil; L, lymphocyte; EOS, eosinophil; NLR, neutrophil to lymphocyte rate; CRP, C-reactive protein; CD3, cluster of differentiation 3; CD4, cluster of differentiation 4; CD8, cluster of differentiation 8; NK, natural killer cell; IL-6, interleukin-6; IL-8, interleukin-8; IL-10, interleukin-10; IL-1β, interleukin-1β; TNF-α, tumor necrosis factor-α; IFN-γ, interferon-γ; SD, standard deviation.

### 3.3 Survival outcome of patients treated with ICIs according to clinical features

**Figure 2:**
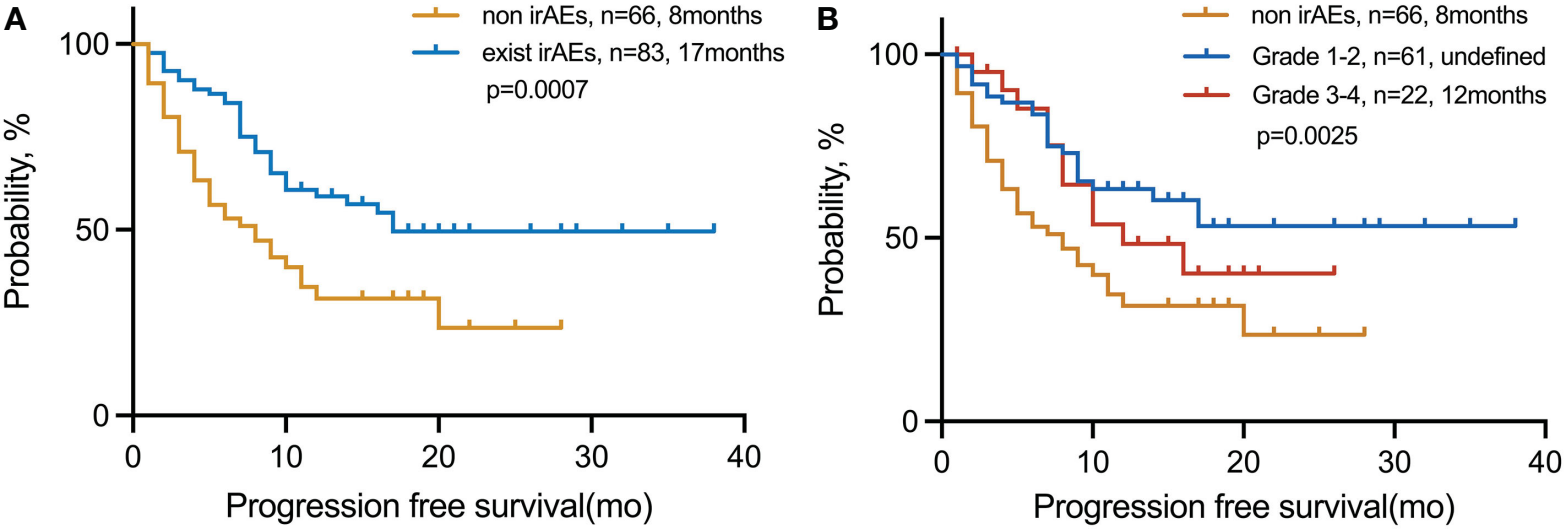
PFS in patients with NSCLC treated with immunotherapy in the **(A)** exist irAE and non-irAE cohorts and **(B)** non-irAE, grades 1–2, and grades 3–4 cohorts. irAEs, immune-related adverse events.

We explored the PFS based on the number of advantageous factors selected from the LT group (**Figure 2**). Of the 149 patients, 83 (55.7%) had irAEs. The median PFS of this group was also significantly longer than that of patients without irAEs, no matter the grade (17 vs. 8 months; *p* = 0.0007) (**Figure 2A**). However, patients with limited irAEs of grades 1–2 were found to have the longest PFS compared with those of higher grades (grades 3–4) and those without irAEs (undefined vs. 12 vs. 8 months; *p* = 0.0025) (**Figure 2B**).

Wild-type NSCLC patients had significantly longer mPFS than EGFR/ALK-mutated patients (20 vs. 4 months; *p* = 0.0009) (**Supplementary Figure S1A**). The mPFS among patients treated with first‐line ICIs were not reached, much longer than those treated with the second-line and above ICIs (undefined vs. 7 months; *p* < 0.0001) (**Supplementary Figure S1B**). Furthermore, in patients with different lines of therapy, grades 1–2 irAEs were the subgroup with the best prognosis (**Supplementary Figure S2**).

### 3.4 Immune-related adverse events

In 83 patients, 128 different irAEs of any grade were reported, including 19 patients with severe irAEs (grade ≥ 3). The commonly seen grades 1–2 irAEs were pneumonia (*n* = 24, 22.0%), endocrinal dysfunctions (*n* = 10, 9.2%), myocarditis (*n* = 10, 9.2%), and rash (*n* = 32, 29.3%). The most common severe irAEs (grade ≥ 3) were also pneumonia (*n* = 13, 68.4%) (**Table 3**).

**Table 3:** Immune-related adverse events according to category and grade.

Category	Grades 1–2	Grades 3–4
All (*N* (%))	109 (100)	19 (100)
Skin (*N* (%))	32 (29.3)	2 (10.5)
Endocrine (*N* (%))	10 (9.2)	0 (0)
Hepatitis (*N* (%))	8 (7.3)	3 (15.8)
Pneumonia (*N* (%))	24 (22.0)	13 (68.4)
Myocarditis (*N* (%))	10 (9.2)	1 (5.3)
Neurologic (*N* (%))	7 (6.4)	0 (0)
Enteritis and diarrhea (*N* (%))	9 (8.3)	0 (0)
Renal (*N* (%))	9 (8.3)	0 (0)

A lower percentage of female patients (30.3% vs. 15.7%; *p* < 0.05), a higher percentage of smokers (50.0% vs. 72.3%; *p* < 0.05), treatment with a PD-1 inhibitor in the first line (33.3% vs. 56.6%; *p* < 0.05), the elderly (62 ± 11 vs. 67 ± 9, *p* < 0.05), higher EOS% (2.3 ± 2.6 vs. 3.2 ± 3.9; *p* < 0.05), and higher IFN-γ (3.8 ± 3.3 vs. 8.2 ± 12.8; *p* < 0.05) were found to be associated with irAEs (**Table 4**).

**Table 4:** Characteristics of patients between whether exist irAEs.

Characteristic	Non-irAEs	Exist irAEs	*p*-value
Number	66	83	–
Age (mean ± SD)	62 ± 11	67 ± 9	0.007^*^
Female (*N* (%))	20 (30.3)	13(15.7)	0.033^*^
Smoker (*N* (%))	33 (50.0)	60 (72.3)	0.005^*^
Squamous (*N* (%))	15 (22.7)	30 (36.1)	0.076
ECOG ≥2 (*N* (%))	6 (9.1)	10 (12.0)	0.562
PD-L1 ≥50% (*N* (%))	18 (43.9)	26 (43.3)	0.955
PD-L1<1% (*N* (%))	11 (26.8)	18 (30.0)	0.729
EGFR/ALK mutation (*N* (%))	12 (18.2)	13 (15.7)	0.865
KRAS mutation (*N* (%))	6 (9.1)	9 (10.8)	0.724
First-line ICIs (*N* (%))	22 (33.3)	47 (56.6)	0.005^*^
Combined treatment (*N* (%))	49 (74.2)	65 (78.3)	0.560
Neu%	69.0 ± 10.0	67.0 ± 10.1	0.309
L%	21.0 ± 8.9	21.0 ± 8.3	0.998
EOS%	2.3 ± 2.6	3.2 ± 3.9	0.025^*^
Neu (×10^9^/L)	5.2 ± 3.3	5.0 ± 2.4	0.994
L (×10^9^/L)	1.4 ± 0.6	1.4 ± 0.6	0.487
NLR	4.6 ± 3.8	4.2 ± 3.1	0.897
EOS (×10^9^/L)	0.2 ± 0.3	0.2 ± 0.4	0.063
CRP (mg/L)	23.2 ± 50.4	33.4 ± 60.2	0.712
CD3	920.3 ± 464.9	908.7 ± 390.0	0.966
CD4	505.0 ± 283.6	531.6 ± 276.9	0.598
CD8	366.2 ± 241.7	348.5 ± 185.5	0.801
NK	327.6 ± 211.5	292.0 ± 200.3	0.302
IL-6 (ng/L)	24.2 ± 43.9	33.6 ± 121.0	0.463
IL-8 (ng/L)	53.5 ± 54.7	59.4 ± 78.6	0.903
IL-10 (ng/L)	4.0 ± 1.8	4.4 ± 4.1	0.369
IL-1β (ng/L)	6.3 ± 6.1	6.4 ± 5.3	0.997
TNF-α (ng/L)	5.9 ± 5.3	4.7 ± 4.0	0.164
IFN-γ (ng/L)	3.8 ± 3.3	8.2 ± 12.8	0.039^*^

^*^p < 0.05.

NLT, non-long-term treatment; LT, long-term treatment; PD-L1, programmed cell death-ligand 1; EGFR, epidermal growth factor receptor; ALK, anaplastic lymphoma kinase; KRAS, Kirsten rat sarcoma viral oncogene homolog; irAEs, immune-related adverse events; ICIs, immune checkpoint inhibitors; Neu, neutrophil; L, lymphocyte; EOS, eosinophil; NLR, neutrophil to lymphocyte rate; CRP, C-reactive protein; CD3, cluster of differentiation 3; CD4, cluster of differentiation 4; CD8, cluster of differentiation 8; NK, natural killer cell; IL-6, interleukin-6; IL-8, interleukin-8; IL-10, interleukin-10; IL-1β, interleukin-1β; TNF-α, tumor necrosis factor-α; IFN-γ, interferon-γ; SD, standard deviation.

However, patients’ ECOG scores and levels of PD-L1 were found to be no different between irAE and non-irAE groups. In our study, neither baseline peripheral-blood eosinophil count nor blood CD3/4/8 level nor cytokine levels (IL-6, IL-8, IL-10, IL-1β, TNF-α) were found to be significantly different between irAE and non-irAE groups (**Table 4**).

### 3.5 Univariate and multivariate analyses of clinical features and biomarkers for irAEs

We performed ROC curve analysis to determine the optimal cutoff value of baseline EOS% and IFN-γ for irAEs (**Supplementary Tables S2**, **S3**): 1.15% for EOS% (AUC = 0.608 (95% CI, 0.515 to 0.701), sensitivity = 79.5%, specificity = 42.2%; *p* = 0.025); 3.75 ng/L for IFN-γ (AUC = 0.642 (95% CI, 0.511 to 0.772), sensitivity = 51.2%, specificity = 81.5%; *p* = 0.047).

**Figure 3:**
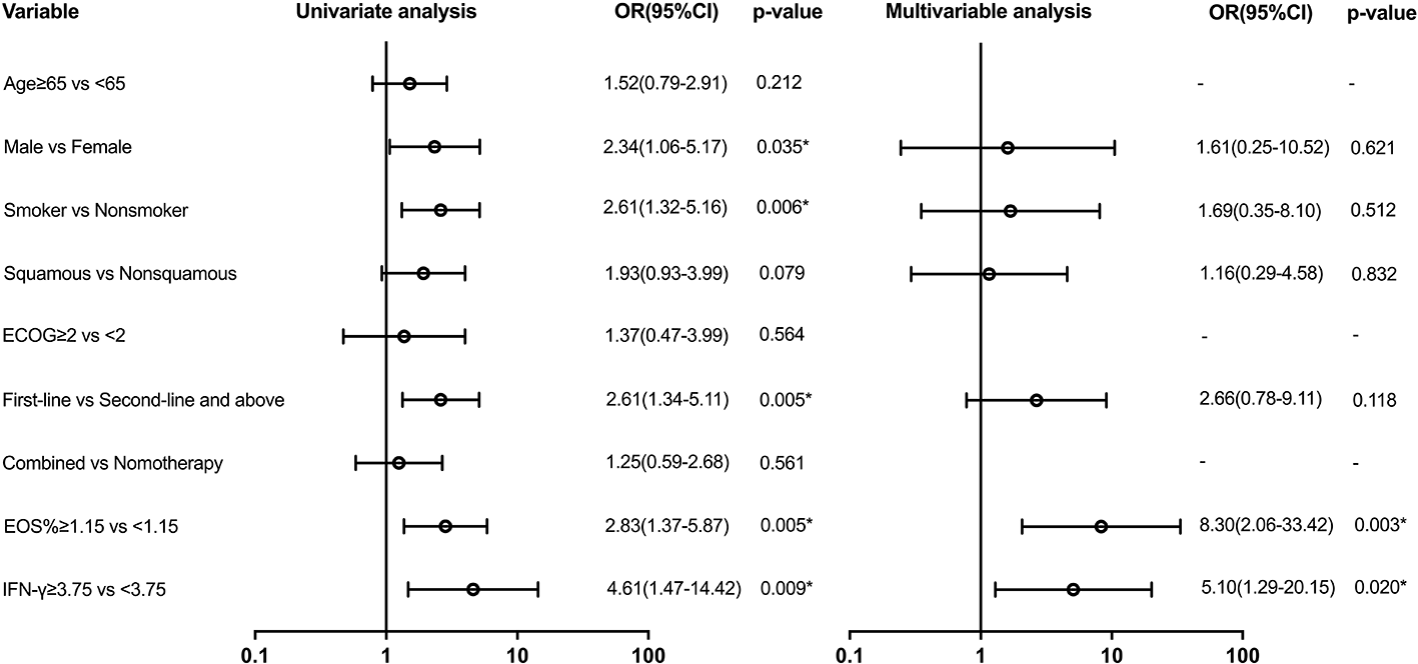
Univariate and multivariable predictors of irAEs. Variables with *p*-value ≤ 0.2 in univariate models were analyzed in the multivariate analysis model. EOS, eosinophil; IFN-γ, interferon-γ; OR, odds ratio; CI, confidence interval. * p<0.05.

Variables with a *p*-value of ≤ 0.2 in univariate models were analyzed in the multivariate analysis model for irAEs (**Figure 3**). Serum biomarkers including EOS% of ≥ 1.15 (HR: 8.30 (95% CI, 2.06 to 33.42); *p* = 0.003) and IFN-γ of ≥ 3.75 (HR: 5.10 (95% CI, 1.29 to 20.15); *p* = 0.02) were found to be predictive for irAEs. However, clinical features including sex, smoking status, pathological type of lung cancer, and first-line immunotherapy were not found valuable in predicting irAEs.

### 3.6 Univariate and multivariate analyses of clinical features and biomarkers for long PFS

**Figure 4:**
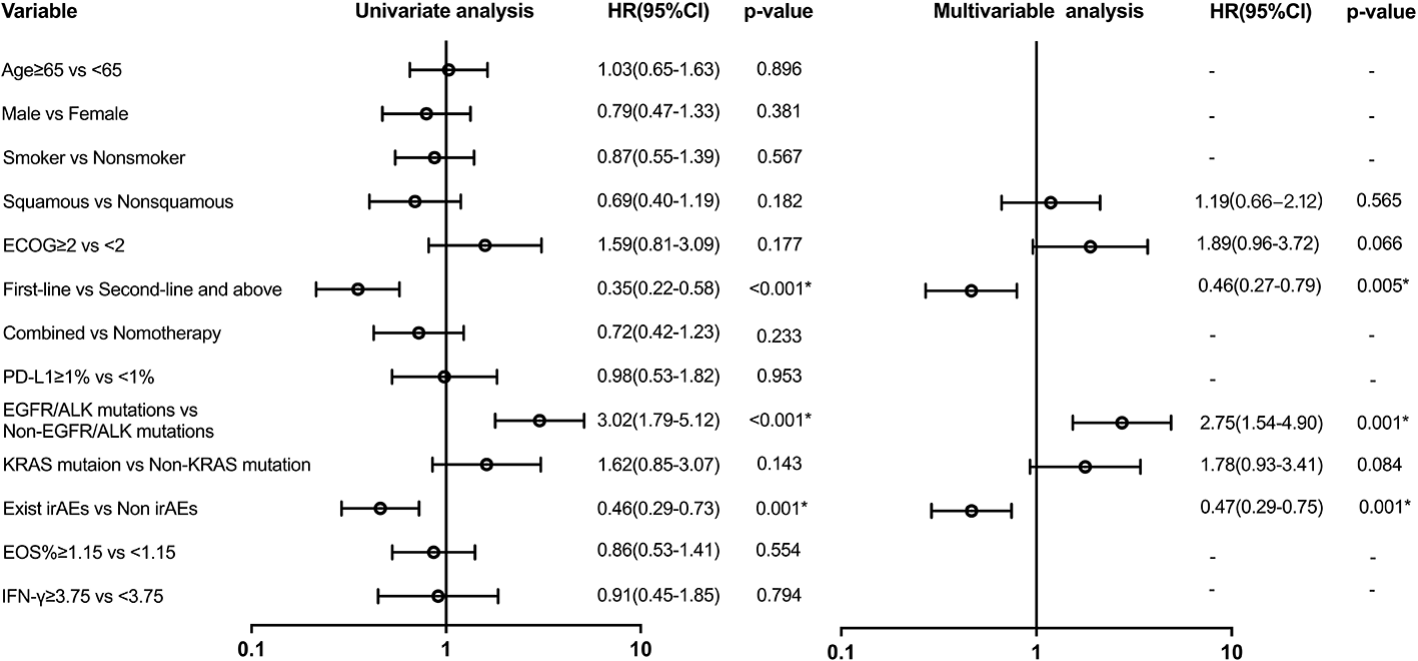
Univariate and multivariable predictors of progression-free survival. Variables with *p*-value ≤ 0.2 in univariate models were analyzed in a multivariate analysis model. EGFR, epidermal growth factor receptor; ALK, anaplastic lymphoma kinase; KRAS, Kirsten rat sarcoma viral oncogene homolog; irAEs, immune-related adverse events; HR, hazard ratio; CI, confidence interval. * p<0.05.

Variables with a *p*-value of ≤ 0.2 in univariate models were analyzed in the multivariate analysis model for PFS (**Figure 4**). There was a difference in PFS favoring first-line ICI recipients when compared with second or above-line ICI recipients (HR: 0.46 (95% CI, 0.27 to 0.79); *p* = 0.005). Patients with EGFR/ALK mutations had shorter PFS than those with non-EGFR/ALK mutations (HR: 2.75 (95% CI, 1.54 to 4.90); *p* = 0.001). There was also a significant difference in PFS in favor of the group with irAEs (HR: 0.47 (95% CI, 0.29 to 0.76); *p* = 0.001). However, EOS% of ≥ 1.15 and IFN-γ of ≥ 3.75 have no difference in PFS.

### 3.7 Validation of EOS% and IFN-γ for predicting the appearance of irAEs

**Figure 5:**
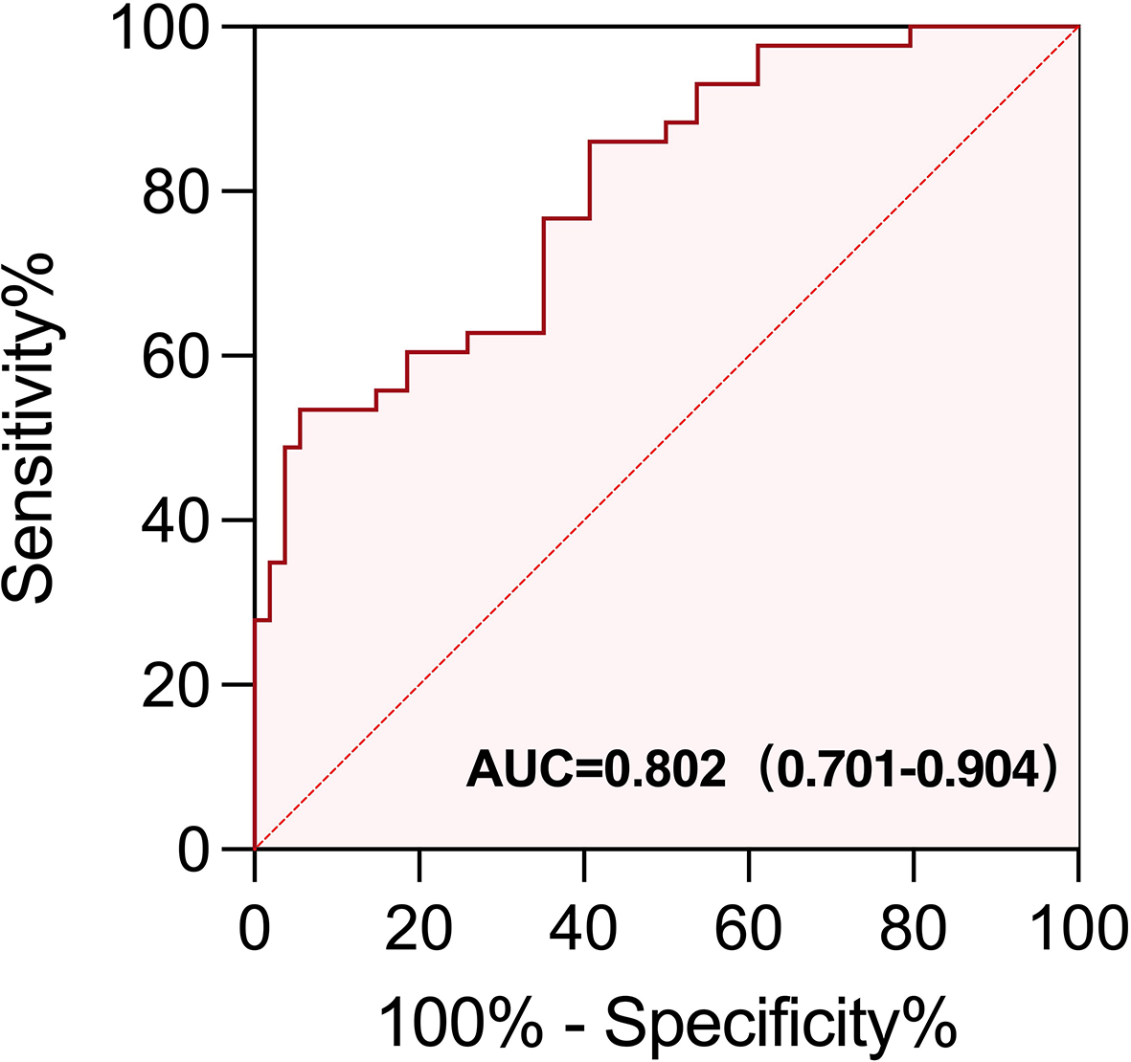
ROC curve of EOS% of ≥ 1.15% combined with IFN-γ of ≥ 3.75 ng/L for predicting the appearance of irAEs in patients treated with immunotherapy. The area under the curve (AUC) of the ROC curve was 0.802 (95% CI, 0.701−0.904; *p* < 0.0001). The sensitivity and specificity were 60.47% and 81.48%, respectively.

To further evaluate the predictive usefulness of serum biomarkers selected from univariate and multifactor models for irAEs, we performed ROC curve analysis to determine the optimal cutoff value of baseline EOS% and IFN-γ. The AUC value demonstrated that EOS% of ≥1.15% combined with IFN-γ of ≥3.75 ng/L had greater predictive power for irAEs (AUC = 0.802 (95% CI, 0.701–0.904)). The sensitivity and specificity were 60.47% and 81.48%, respectively (**Figure 5**).

### 3.8 Multisystem irAEs and corticosteroid treatment

**Figure 6:**
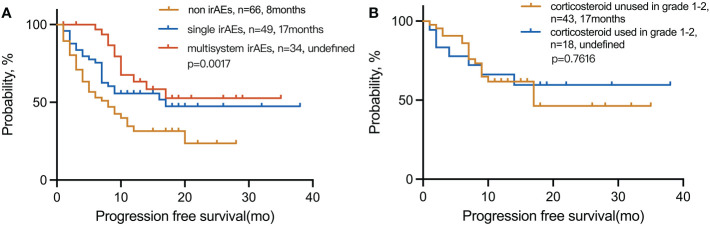
Kaplan–Meier curves for PFS in **(A)** patients with multisystem irAEs compared to patients with single irAEs and non-irAEs and **(B)** patients with irAEs in grades 1–2 used corticosteroid compared to unused. irAEs, immune-related adverse events.

The mPFS for multiple-organ system irAEs vs. single-organ irAE vs. non-irAE groups was (undefined vs. 17 and 8 months; *p* = 0.0017), respectively. The 1-year PFS rate for multisystem vs. single vs. non-irAE groups was 63%, 56%, and 31%, respectively (**Figure 6**). In the presence of grades 1–2 irAEs, the use of corticosteroids has no significant effect on the efficacy of immunotherapy.

## 4 Discussion

The heterogeneous nature of patients makes ICI treatment ineffective for all of them. Noninvasive measurement to predict treatment prognosis and to improve treatment management is one of the clinical needs. In the first phase, we try to identify clinical features and serum biomarkers in those advanced NSCLC patients who benefit from ICI treatment. We also found that the positive effect of ICI treatment, such as longer PFS, was associated with the appearance of irAEs. An evaluation of the relationship between irAEs and immunotherapy efficacy was conducted retrospectively. To identify the high-risk patients of irAEs in the real world, EOS% of ≥1.15% and IFN-γ of ≥3.75 ng/L were considered dependent risk factors in univariate and multivariate logistic regression analyses.

Immunotherapy made a breakthrough in the first-line treatment of NSCLC patients, and immunotherapy combinations further expanded potential beneficiaries regardless of PD-L1 expression (1, 11). Our study also found PFS benefit from first-line anti-PD-(L)1 treatment in unselected patients. Many clinical trials, such as KEYNOTE-189 (12), KEYNOTE-407 (13), and KEYNOTE-042 (14), have shown that first-line immunotherapy substantially improved OS and PFS. At present, first-line immunotherapy combined with chemotherapy has become the standard treatment strategy for EGFR and ALK-negative advanced NSCLC patients. Our study found that in patients with EGFR or ALK mutations, PFS was significantly shorter than in wide-type patients (20 m vs. 4 m). Those patients with driven mutations usually received targeted therapy first line, and although they achieved relatively good efficacy, they still needed a change in medication strategy when they progressed or relapsed after treatment. Whether patients with EGFR or ALK mutations can receive immunotherapy is still controversial (15–17).

Intriguingly, irAEs were observed in 52 of 109 (47.7%) NLT groups vs. 31 of 40 (77.5%) LT groups (*p* = 0.001). It appears that irAEs are closely related to clinical outcomes. According to the previous study, the occurrence of irAEs is positively correlated with clinical benefits (18). One mechanism is that activated T cells and increased cytokines induced by ICIs target both tumors and host organs with common antigens, which also shows that ICIs play a role in immune enhancement (19). On the other hand, when the effect of a double-edged sword occurs, it is often due to the impact of inflammatory progression and corticosteroid treatment. Serious adverse events caused by immunotherapy will have an impact on patients’ basic situation, leading to suspension or even termination of antitumor therapy. In addition, long-term corticosteroid therapy for patients with severe irAEs may have an effect on prognosis. In our study, irAEs were found to be an independent predictor of PFS (HR: 0.47 (95% CI, 0.29 to 0.75); *p* = 0.001). Consistent with previous studies, patients who experienced irAEs had a significantly higher ORR and a longer PFS than patients who did not (20–22).

Our study suggested that the most commonly involved organ was pneumonia (37/83, 44.6%), and a proportion of pneumonia irAEs (13/83, 15.7%) were rated above grade 3. Another commonly involved organ was the skin (34/83, 41.0%), but only a small proportion of skin irAEs (2/83, 2.4%) were rated above grade 3. Severe irAEs can lead to interruption of immunotherapy and often have a lower median OS (23). A meta-analysis of ICI-related fatal irAEs found that the fatality rate associated with pneumonitis was 35% (115/333) (24). However, our study found that even in the presence of grades 3–4 irAEs, the prognosis of patients is still better in the absence of irAEs. It has also been observed that individual patients who progress to multiple-organ system irAEs are benefiting from ICI treatment, which gives clinicians confidence when encountering irAEs.

The investigation of irAE predictive markers is still ongoing in order to identify high-risk groups in advance. We found that baseline increases in peripheral-blood EOS% and IFN-γ were significantly related to irAEs. A retrospective study found that absolute eosinophil count was shown to be linked with the appearance of ICI-pneumonitis in patients receiving ICI-based therapy (10). Previous studies has shown that eosinophils act as regulators or effector cells in tumor rejection by regulating the tumor microenvironment, such as attracting tumor-specific CD8+ T cells and promoting the maturation of several immune cells (25). The IFN-γ axis is an indispensable link in ICI treatment because it enhances tumor immunogenicity (26–28). Some studies suggested that active IFN-γ signaling triggers apoptosis and cell cycle arrest in lung cancer cells, explaining the mechanism of its antitumor effect (29, 30). Nevertheless, high expression of EOS% and IFN-γ can induce chemokine increases and magnify inflammation; at this time, irAE may be a type of collateral damage (31, 32). In the previous study, NSCLC patients with high expression of IFN-γ exhibited longer PFS and OS with immunotherapy (33). However, there was no association between the peripheral-blood markers and PFS benefit in our study. One possible reason for this is that the current study’s patients population was insufficient. Second, patients with a high EOS% and IFN-γ had a higher risk of irAEs, which may indirectly influence the PFS. Pay attention to high-risk patients whose baseline peripheral-blood EOS% of ≥ 1.15% and IFN-γ of ≥3.75 ng/L may help in balancing therapeutic benefit and treatment-related toxicity.

The treatment of irAEs is largely empirical. There is still controversy over whether corticosteroids are needed for grades 1–2-irAEs. A retrospective analysis showed that corticosteroid treatment for irAEs does not affect the clinical outcomes of patients with melanoma (34). Nevertheless, another study showed that patients who received corticosteroid treatment during ICIs had a significantly poor prognosis (35). In our study, we analyzed the survival curve of grades 1–2 irAE patients with or without corticosteroid treatment and found that corticosteroid treatment does not affect the immunotherapy efficacy and prognosis of these patients. Thus, practitioners and patients should consider using corticosteroids as appropriate to prevent aggravation of irAEs.

ICI drugs are widely used, and many existing large clinical trials are designed for drug efficacy, but there are few clinical trials for irAEs, so research on irAEs is in urgent need of real-world data analysis. We found that in the current clinical treatment of lung cancer in China, there are many different types of immune checkpoint inhibitors, including imported and domestic drugs, with vastly different therapeutic regimens from those found elsewhere. As a result, our study objectively reflects the real-world experience of ICI therapy in Chinese lung cancer patients. irAEs are still a field worth studying, and our retrospective study showed that irAEs are not fatal, and that patients can still have a good prognosis with early detection and treatment.

This study has several limitations. First, the total number of patients enrolled was small. Second, only a small proportion of patients underwent molecular pathological analysis, which may have an impact on the efficacy of ICIs.

## 5 Conclusions

Immunotherapy showed promising clinical outcomes as a first-line treatment for all patients with advanced NSCLC. Clinical factors such as the lack of driven gene mutations and the appearance of irAEs were independent predictive factors for PFS. EOS% of ≥1.15% and IFN-γ of ≥3.75 ng/L were considered peripheral-blood markers for irAEs.

## Data availability statement

The original contributions presented in the study are included in the article/**Supplementary Material**. Further inquiries can be directed to the corresponding authors.

## Ethics statement

The studies involving human participants were reviewed and approved by Institutional Review Board of Shanghai Rui Jin hospital (2019–72). Written informed consent for participation was not required for this study in accordance with the national legislation and the institutional requirements.

## Author contributions

Conceptualization: YG and XW. Data curation: W-TH, QZ, ZZ and XH. Formal analysis: W-TH, QZ, ZZ and XH. Funding acquisition: MZ and YG. Methodology: W-TH, MZ and YG. Software: W-TH, QZ, ZZ and XH. Supervision: XW. Visualization: MZ. Writing—original draft. W-TH and XW. Writing—review, and editing: MZ, YG and XW. All authors contributed to the article and approved the submitted version.
