# Supplementary material for: Eosinophil and IFN-γ associated with immune-related adverse events as prognostic markers in patients with non-small cell lung cancer treated with immunotherapy
Source: Front Immunol. 2023 Mar 6;14:1112409. doi: 10.3389/fimmu.2023.1112409 (PMC10025375; doi:10.3389/fimmu.2023.1112409)
Supplement: Supplementary file 1 [file DataSheet_1.docx]

Supplementary Material

A cohort study of clinically relevant prognostic and predictive markers for immune-checkpoint-inhibitor (ICI) therapy in non-small cell lung cancer (NSCLC)

# Supplementary Figures and Tables

## Supplementary Figures


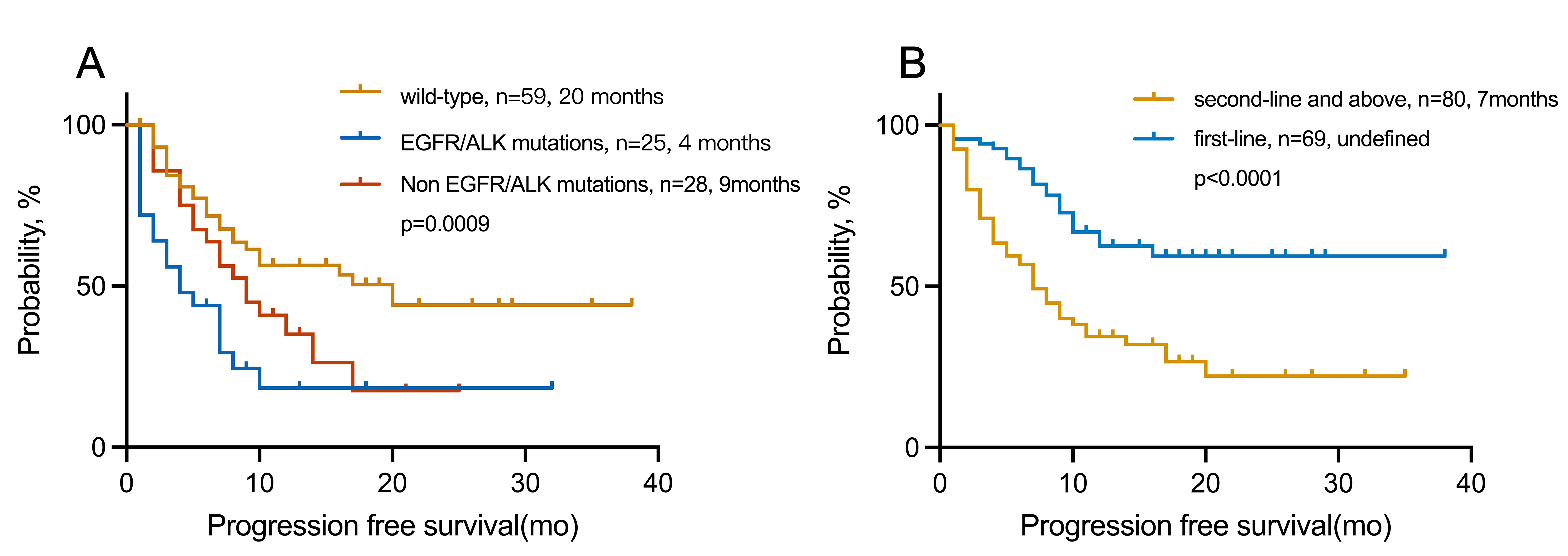


**Supplementary Figure S1** PFS in patients with NSCLC treated with immunotherapy in the (A) wild-type, EGFR/ALK mutations and Non EGFR/ALK mutations cohorts (B) first-line and second-line and above cohorts.

**Abbreviations:** EGFR, epidermal growth factor receptor; ALK, anaplastic lymphoma kinase.


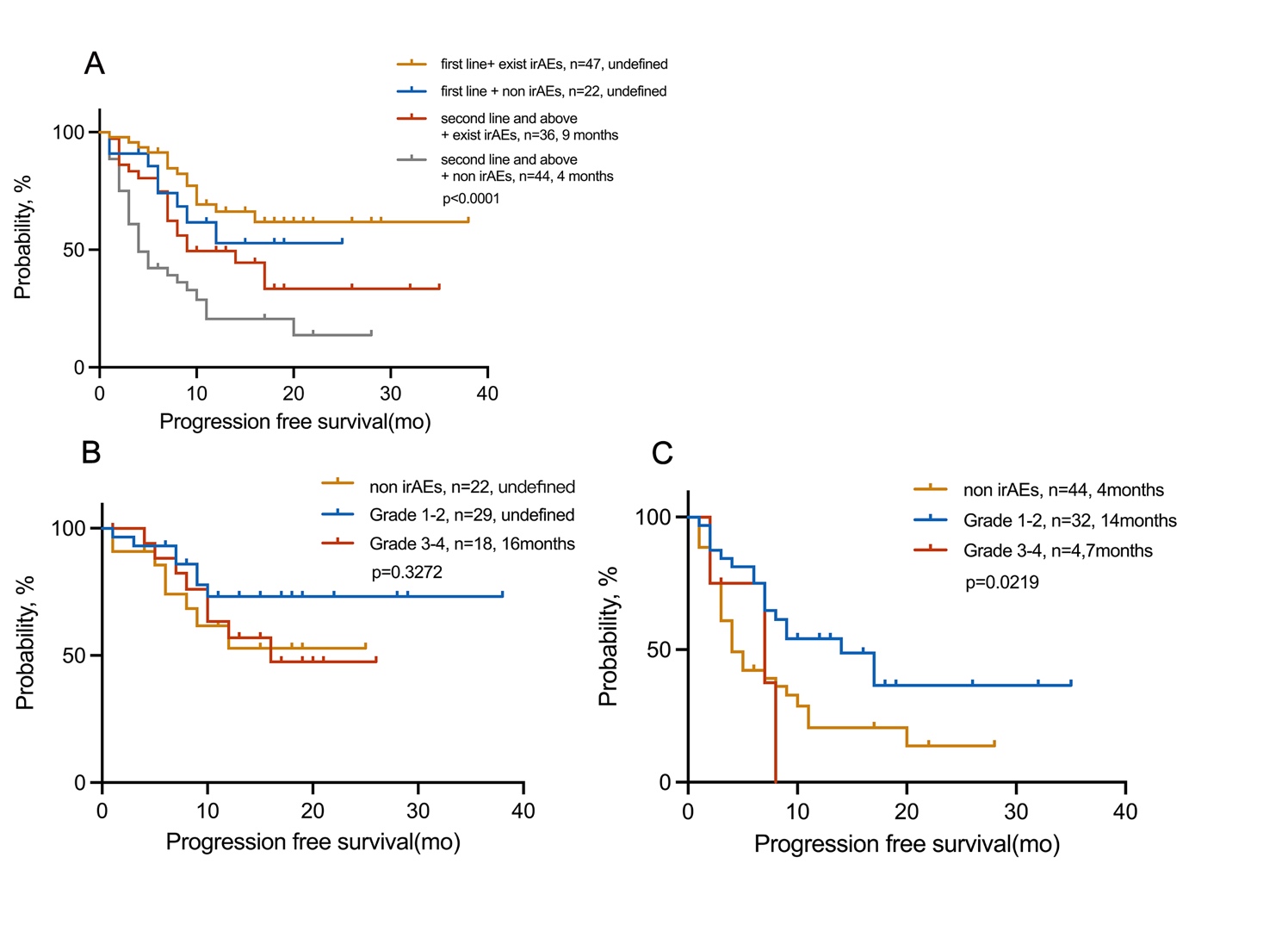


**Supplementary Figure S2** PFS in patients with NSCLC treated with immunotherapy in the (A) exist irAEs and non irAEs cohorts in different lines; (B) non irAEs, 1-2 grade and 3-4 grade cohorts in first line therapy patients; (C) non irAEs, 1-2 grade and 3-4 grade cohorts in second line and above therapy patients.

## Supplementary Tables

| **Supplementary Table S1** Immune checkpoint inhibitor therapy regimens | |
| --- | --- |
| Therapeutic schedule | Total, N (%) |
| Monotherapy | 35 (23.5) |
| Combination therapy | 114 (76.5) |
| +Chemotherapy | 66 (44.3) |
| +Anti-angiogenic drugs | 23 (15.4) |
| + Anti-angiogenic and chemotherapy | 25 (16.8) |

| **Supplementary Table S2** Coordinate of ROC curve of EOS% for predicting the irAEs | | |
| --- | --- | --- |
| cut-off value | sensitivity | specificity |
| -1 | 1 | 0 |
| 0.05 | 0.976 | 0.016 |
| 0.15 | 0.964 | 0.047 |
| 0.235 | 0.964 | 0.062 |
| 0.285 | 0.964 | 0.078 |
| 0.35 | 0.94 | 0.141 |
| 0.45 | 0.928 | 0.141 |
| 0.55 | 0.904 | 0.219 |
| 0.65 | 0.904 | 0.266 |
| 0.75 | 0.892 | 0.328 |
| 0.85 | 0.88 | 0.328 |
| 0.95 | 0.843 | 0.344 |
| 1.05 | 0.819 | 0.391 |
| 1.15 | 0.795 | 0.422 |
| 1.25 | 0.771 | 0.422 |
| 1.35 | 0.747 | 0.437 |
| 1.45 | 0.735 | 0.453 |
| 1.55 | 0.699 | 0.5 |
| 1.65 | 0.663 | 0.5 |
| 1.75 | 0.59 | 0.547 |
| 1.85 | 0.53 | 0.594 |
| 1.95 | 0.494 | 0.594 |
| 2.05 | 0.482 | 0.594 |
| 2.15 | 0.482 | 0.609 |
| 2.25 | 0.47 | 0.625 |
| 2.35 | 0.434 | 0.656 |
| 2.45 | 0.422 | 0.687 |
| 2.55 | 0.422 | 0.703 |
| 2.65 | 0.386 | 0.719 |
| 2.75 | 0.386 | 0.734 |
| 2.85 | 0.373 | 0.734 |
| 2.95 | 0.361 | 0.75 |
| 3.05 | 0.325 | 0.75 |
| 3.15 | 0.301 | 0.766 |
| 3.3 | 0.289 | 0.766 |
| 3.45 | 0.289 | 0.781 |
| 3.55 | 0.277 | 0.797 |
| 3.7 | 0.265 | 0.797 |
| 3.85 | 0.253 | 0.812 |
| 4.05 | 0.253 | 0.828 |
| 4.25 | 0.217 | 0.859 |
| 4.35 | 0.205 | 0.859 |
| 4.45 | 0.193 | 0.859 |
| 4.55 | 0.181 | 0.891 |
| 4.75 | 0.157 | 0.891 |
| 5 | 0.133 | 0.906 |
| 5.15 | 0.12 | 0.922 |
| 5.5 | 0.12 | 0.937 |
| 6.15 | 0.108 | 0.953 |
| 6.8 | 0.096 | 0.953 |
| 7.25 | 0.084 | 0.953 |
| 7.55 | 0.084 | 0.969 |
| 7.85 | 0.072 | 0.969 |
| 9.45 | 0.06 | 0.984 |
| 11.05 | 0.048 | 0.984 |
| 11.65 | 0.036 | 0.984 |
| 12.3 | 0.024 | 0.984 |
| 14.85 | 0.012 | 0.984 |
| 23.05 | 0.012 | 1 |
| 29.9 | 0 | 1 |
| Note: The area under the curve (AUC) of the ROC curve was 0.608 (95% CI 0.515−0.701; p=0.025). The cut-off value is 1.15%, the sensitivity and specificity were 79.5% and 42.2%, respectively. | | |

| **Supplementary Table S3** Coordinate of ROC curve of IFN-γ for predicting the irAEs | | |
| --- | --- | --- |
| cut-off value | sensitivity | specificity |
| 1.4 | 1 | 0 |
| 2.45 | 0.651 | 0.519 |
| 2.6 | 0.605 | 0.593 |
| 2.8 | 0.581 | 0.593 |
| 2.95 | 0.558 | 0.593 |
| 3.05 | 0.558 | 0.667 |
| 3.25 | 0.558 | 0.741 |
| 3.45 | 0.535 | 0.741 |
| 3.6 | 0.535 | 0.778 |
| 3.75 | 0.512 | 0.815 |
| 3.85 | 0.465 | 0.815 |
| 4 | 0.442 | 0.815 |
| 4.25 | 0.442 | 0.852 |
| 4.55 | 0.372 | 0.852 |
| 4.85 | 0.326 | 0.852 |
| 5.1 | 0.256 | 0.852 |
| 5.75 | 0.256 | 0.889 |
| 6.7 | 0.233 | 0.926 |
| 7.6 | 0.209 | 0.926 |
| 8.3 | 0.186 | 0.926 |
| 10.4 | 0.163 | 0.926 |
| 13.05 | 0.14 | 0.926 |
| 14.9 | 0.14 | 0.963 |
| 16.05 | 0.14 | 1 |
| 17.2 | 0.116 | 1 |
| 25.5 | 0.093 | 1 |
| 33.55 | 0.07 | 1 |
| 36.95 | 0.047 | 1 |
| 53.7 | 0.023 | 1 |
| 68.9 | 0 | 1 |
| **Note:** The area under the curve (AUC) of the ROC curve was 0.642 (95% CI 0.511−0.772; p=0.025). The cut-off value is 3.75, the sensitivity and specificity were 51.2% and 81.5%, respectively. | | |
